# Renal and Glucose-Lowering Effects of Empagliflozin and Dapagliflozin in Different Chronic Kidney Disease Stages

**DOI:** 10.3389/fendo.2019.00820

**Published:** 2019-11-22

**Authors:** Yi-Hsuan Lin, Yu-Yao Huang, Sheng-Hwu Hsieh, Jui-Hung Sun, Szu-Tah Chen, Chia-Hung Lin

**Affiliations:** ^1^Division of Endocrinology and Metabolism, Department of Internal Medicine, Chang Gung Memorial Hospital, Linkou, Taiwan; ^2^Department of Medical Nutrition Therapy, Chang Gung Memorial Hospital, Linkou, Taiwan; ^3^Department of Chinese Medicine, College of Medicine, Chang Gung University, Taoyuan, Taiwan

**Keywords:** sodium–glucose cotransporter 2 inhibitors, renal function, acute kidney injury, Chang Gung Research Database, glucose control

## Abstract

**Objective:** The objective of this study was to investigate the effects of sodium-glucose cotransporter 2 (SGLT2) inhibitors on renal function in different stages of chronic kidney disease (CKD).

**Design and Methods:** We conducted a retrospective cohort study using longitudinal claims data from May 2016–December 2017 from the Chang Gung Research Database. Patients who used one of the three types of SGLT2 inhibitor available at Chang Gung Memorial Hospital, namely empagliflozin 10 mg/tab (Empa10), empagliflozin 25 mg/tab (Empa25), and dapagliflozin 10 mg/tab (Dapa), were included, with the same number of matched non-users. Analysis of variance was used for continuous variables and the chi-square test was applied for categorical variables. Differences in data between two groups were analyzed using an independent *t*-test, and the basic data before and after treatment were analyzed using generalized estimating equation (GEE). The association among renal function changes was analyzed using a Cox proportional hazards model, with the results presented as unadjusted hazard ratios (HRs) with 95% confidence intervals (95% CIs).

**Results:** Among the 7,624 SGLT2 inhibitor users, 1,696 patients used Empa10, 2,654 used Empa25, and 3,274 used Dapa. Compared with non-users, dapagliflozin had the lowest risk of estimated glomerular filtration rate (eGFR) decrease over 40% from baseline within 1 year (HR 0.36, 95% CI 0.25–0.51). By using the ICD-10-CM code N179, the acute kidney injury (AKI)-related hospitalization rate was lower in Empa10 and Dapa users than in non-users (HR 0.65, 95% CI 0.49–0.86).

**Conclusion:** Lower risk of eGFR decrease over 40% and AKI-related hospitalization was found in all SGLT2 inhibitor users across the different CKD stages.

## Introduction

The sodium–glucose cotransporter2 (SGLT2) inhibitor belongs to the newest class of antidiabetic drugs. It inhibits SGLT2 transporter in the kidney, increases urinary glucose excretion, and reduces the serum glucose level (1). Although the initial purpose of using this medication was to achieve better glycemic control, which can decrease glycosylated hemoglobulin (HbA1c) about 0.5–0.8% (2), several additional benefits were noted in clinical trials, including decreases in body weight, blood pressure, and the rate of hospitalization for heart failure and death from cardiovascular causes (3, 4). Moreover, because this medication's mechanism of action depends on the kidneys, patients who receive SGLT2 inhibitors must have a certain level of renal filtration function (5). Therefore, whether SGLT-2 inhibitors have adverse effects on renal function and lead to more renal injury events must be determined. Large-scale clinical trials of SGLT2 inhibitors, including the EMPA-REG and CANVAS studies, have revealed that although the initial eGFR decreases rapidly, there was better eGFR of SGLT2 inhibitors users after follow up 192 weeks (4–6). And the DECLARE study also presented that dapagliflozin had lower cumulative incidence of ≥40% decrease in eGFR <60 mL/min/1.73 m^2^ of body-surface area, new end-stage renal disease, or death from renal or cardiovascular causes than placebo (7). Furthermore, there was considerable reduction of urine albumin/creatinine ratio (UACR) on SGLT2 inhibitor users compared with placebo users after a certain follow-up period, especially those who had CKD (8, 9). However, because of limited real-world data, whether SGLT2 inhibitors lead to changes in renal filtration function remains debatable. Although some studies have reported that SGLT2 inhibitors had no adverse effect on renal function (10) or even protection effects (11, 12), some studies have reported different findings. According to Perlman et al., SGLT2 inhibitors might be related to an increase in acute renal failure events (13), a result which is contradicted by clinical trials. In addition, limited data indicate that SGLT2 inhibitors cause AKI and related hospitalization. Furthermore, more clinical data and research are necessary to elucidate whether the two most widely used SGLT2 inhibitors, namely empagliflozin (Empa) and dapa, differ in their effect.

We therefore conducted a retrospective cohort study using longitudinal claims data from the Chang Gung Research Database (CGRD), a de-identified database derived from the medical records of Chang Gung Memorial Hospital (CGMH), to focus on renal function change, acute renal deterioration events and glucose-lowering effect. Specifically, the aims of this study was to determine whether using SGLT2 inhibitors alone and other drugs had a beneficial effect in different chronic kidney disease stage patients with diabetes.

## Methods

### Data Source

We conducted a retrospective cohort study using longitudinal claims data for May 2016–December 2017 from CGRD, a de-identified database derived from medical records of CGMH. CGMH, founded in 1976, is one of the largest medical institutions in Taiwan, with 7 hospitals extending from northeast to southern Taiwan. This study was approved by the CGMH Institutional Review Board.

### Study Population

We identified 70,461 patients with type 2 diabetes mellitus and 7,624 patients (age ≥ 20 years) who used one of SGLT2 inhibitors available at CGMH, namely empagliflozin 10 mg/tab (Empa10), empagliflozin 25 mg/tab (Empa25), and dapagliflozin 10 mg/tab (Dapa) defined as SGLT2 inhibitor users, with the same number of propensity score matched non-users during January 2016–December 2017. The matched SGLT-2 inhibitor non-users were chosen in those who have the diagnosis of diabetes but no SGLT2 inhibitor usage in their medical records in CGRD database. The index date was defined as the first date of therapy during the study period. Patients receiving a SGLT2 inhibitor for <7 days were excluded. Some patients who were prescribed with Empa10 initially then change to Empa25 were grouped to Empa25 if the use of Empa25 was over 3-months continuously. The following biochemistry data were collected before the first medication use and the closest data before December 2017 at least 28 days after medication: serum creatinine (Cre), HbA1c, preprandial (AC) and peak postprandial (PC) blood glucose, urine albumin (Alb), urine Cre, and UACR. Moreover, we analyzed the effect of SGLT2 inhibitors as well as other anti-diabetic drugs, including acarbose (50 and 100 mg), dipeptidyl peptidase-4 (DPP-4) inhibitors (sitagliptin, vildagliptin, saxagliptin, linagliptin, and alogliptin), insulin (glargin, detemir, aspart, lispro, glulisine, NovoMix 30/50, and Humalog Mix 25/50), glucose-like peptide-1 (GLP-1) agonists (exenatide, liraglutide, and dulaglutide), sulfonylurea (SU) (glimepiride, gliclazide, and glyburide), glinide (repaglinide), and metformin. An acute renal deterioration event was identified as eGFR decrease over 40% from baseline (7, 14) or AKI-related hospitalization within 1 year. The AKI-related hospitalization was identified with International Classification of Diseases, Tenth Revision, Clinical Modification (ICD-10-CM), an international medical diagnosis code, code N179. The eGFR level was calculated by the CKD-EPI (Epidemiology Collaboration) equation (15). Patients treated with SGLT2 inhibitors were matched to patients treated with other antidiabetic drugs through propensity score matching in a 1:1 ratio on the basis of age, sex, baseline serum creatinine and A1C levels, use of antidiabetic drugs (DPP-4, insulin, GLP-1, SU, glinide, and metformin), statins, antihypertensive drugs [Angiotensin II receptor blocker (ARB), angiotensin converting enzyme inhibitor (ACEI), loop diuretics, thiazides, aldosterone antagonist, beta blocker, and calcium channel blocker (CCB)], and comorbidities [Alzheimer's disease (AD), hypertension, hyperlipidemia, cerebrovascular accident (CVA), ischemia, hemorrhage, coronary artery disease (CAD), myocardial infarction, ischemic heart disease, and heart failure]. The baseline characteristics of those accounted confounding factors were matched and present in the Supplementary Table 1.

### Main Outcome Measures

The primary outcome of this study was renal function changes, including acute renal deterioration (eGFR decrease over 40% within 1 year) and AKI-related hospitalization during the follow-up period. The secondary outcome was the difference of HbA1c changes between SGLT2 inhibitor users and non-users.

### Statistical Analysis

The baseline characteristics of the three treatment groups (Empa10, Empa25, and Dapa) were compared using analysis of variance for continuous variables and the chi-square test for categorical variables. Differences in data between two groups were analyzed using an independent *t*-test, and the basic data before and after treatment were analyzed using GEE. The association among renal function changes was analyzed using a Cox proportional hazards model, with the results presented as unadjusted HRs with 95% CIs. To reduce differences in baseline characteristics among the groups, we performed propensity score matching. The baseline characteristics and Cox proportional hazards model for adjusted HRs and 95% CIs are also presented for matched pairs. All statistical analyses were performed using SAS statistical software (version 9.4; SAS Institute, Inc., Cary, NC, USA). A two-sided *p* < 0.05 was considered statistically significant.

## Results

### Study Population Characteristics

From May 1, 2016 to December 31, 2017, a total of 70,461 individuals with diabetes mellitus were registered in the CGRD. Among these patients, 7,624 patients were included as SGLT2 inhibitor users, with the same number of patients matched as non-users. Demographic characteristics such as sex and age as well as changes in biochemistry data before the first medication use and the closest data before December 2017 are summarized in Table 1.

**Table 1:** Characteristics of the study population of Sodium glucose co-transporter 2 inhibitor users and non-users.

**Basic statistic**					**SGLT2i vs. non-users**	**SGLT2i vs. non-users**
	**SGLT2i**	**Diff (1st-last)**	**Matched non-users**	**Diff (1st-last)**	***p*****-value**	***p*****-value Diff (1st-last)**
No.	7,624		7,624			
Age, year	61.9 ± 11.6		61.5 ± 13.		0.061	
Sex (female), %	41.9%		42.2%		0.768	
Cre (first before drug) (mg/dL)	0.895 ± 0.354	−0.035	0.889 ± 0.382	−0.054	0.361	<0.001^*^
Cre (last) (mg/dL)	0.930 ± 0.432		0.943 ± 0.502		0.096	
eGFR (first before drug) (mL/min/1.73 m^2^)	92.692 ± 31.425	2.554	96.798 ± 38.059	4.898	<0.001^*^	<0.001^*^
eGFR (last) (mL/min/1.73 m^2^)	90.138 ± 32.636		91.900 ± 35.889		0.002	
HbA1c (first before drug) (%, mmol/mol)	8.890 ± 1.620 (73.839 ± 17.704)	0.706 (7.718)	8.985 ± 2.266 (74.690 ± 24.771)	0.991 (10.829)	0.015 (<0.015^*^)	<0.001^*^
HbA1c (last) (%, mmol/mol)	8.201 ± 1.407 (66.121 ± 15.376)		7.994 ± 1.729 (63.861 ± 18.905)		<0.001^*^	
UACR (first before drug) (mg/g)	45.445 ± 75.175	−7.778	107.529 ± 542.564	−0.868	0.336	0.391
UACR (last) (mg/g)	53.223 ± 95.205		108.397 ± 542.692		0.399	

*Age, Cre, eGFR, HbA1c, and UACR presented as mean ± SD*.

*SGLT-2i, Sodium-glucose co-transporter 2 inhibitor; Diff, difference; Cre, creatinine; eGFR, estimated Glomerular filtration rate; HbA1c, Glycated hemoglobin; UACR, urine albumin to creatinine ratio*.

* *p < 0.05*.

### Comparison Between SGLT2 Inhibitor Users and Non-users

**Figure 1:**
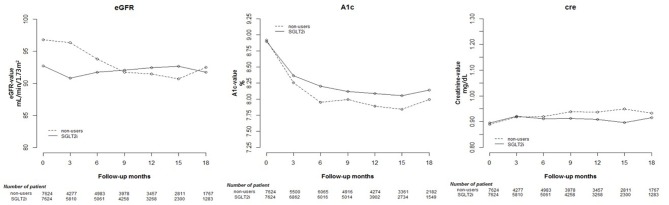
Change in estimated glomerular filtration rate (eGFR), and glycated hemoglobin (HbA1c), and creatinine (Cre) levels in users of sodium–glucose cotransporter 2 (SGLT2) inhibitors and other drugs.

HbA1c decreased after at least 12 weeks in both SGLT2 inhibitor users and non-users Regarding renal function parameters, elevated Cre and reduced eGFR were more prominent in non-users than in SGLT2 inhibitor users. The results are shown in Table 1 and Supplementary Table 2. The changes in Cre and HbA1c levels and eGFR are shown in Figure 1, indicating significant differences in changes between the two groups (*p* < 0.001).

### Comparisons Among Different SGLT2 Inhibitor User Groups

Considering SGLT2 inhibitor users according to the different drugs and dosages used, 1,696 patients used Empa10, 2,654 patients used Empa25, and 3,274 patients used Dapa. Demographic characteristics such as sex and age as well as changes in biochemistry data before the first medication use and the closest data before December 2017 are summarized in Table 2 and Supplementary Table 3.

**Table 2:** Characteristics of the study population of three kinds of Sodium glucose co-transporter 2 inhibitor.

**Basic statistic**	**Empagliflozin (10 mg)**	**Diff (1st-last)**	**Empaglifozin (25 mg)**	**Diff (1st-last)**	**Dapagliflozin (10 mg)**	**Diff (1st-last)**	**Empa10 vs. Empa25 vs. Dapa *p*-value**	**Empa10 vs. Empa25 diff (varl-varf) *p*-value**	**Empa10 vs. Dapa diff (varl-varf) *p*-value**	**Empa25 vs. Dapa diff (varl-varf) *p*-value**
*N*	1,696		2,654		3,274					
Age, year	63.2 ± 11.9		62.0 ± 11.7		61.2 ± 11.4		0.019^a^^,^^b^^,^^c^			
Sex (female), %	42.2%		39.9%		43.5%		<0.001^b^			
Cre (first before drug) (mg/dL)	0.925 ± 0.421	−0.029	0.942 ± 0.402	−0.018	0.841 ± 0.251	−0.011	<0.001^b^^,^^c^	0.165	0.010^*^	0.163
Cre (last) (mg/dL)	0.954 ± 0.543		0.960 ± 0.492		0.852 ± 0.296		<0.001^b^^,^^c^			
eGFR (first before drug) (mL/min/1.73 m^2^)	91.033 ± 33.474	0.628	89.551 ± 32.985	0.429	96.097 ± 28.581	−0.11	<0.001^b^^,^^c^	0.689	0.145	0.217
eGFR (last) (mL/min/1.73 m^2^)	90.405 ± 34.715		89.122 ± 33.096		96.207 ± 32.902		<0.001^b^^,^^c^			
HbA1c (first before drug) (%, mmol/mol)	8.731 ± 1.621 (71.913 ± 17.721)	0.706 (7.714)	8.864 ± 1.724 (73.375 ± 18.848)	0.639 (6.991)	8.976 ± 1.553 (74.596 ± 16.980)	0.785 (8.578)	<0.001^a^^,^^b^^,^^c^	0.122	0.057	<0.001^*^
HbA1c (last) (%, mmol/mol)	8.025 ± 1.418 (64.199 ± 15.506)		8.225 ± 1.446 (66.384 ± 15.806)		8.191 ± 1.347 (66.018 ± 14.726)		<0.001^a^^,^^c^			
UACR (first before drug) (mg/dL)	5.675 ± 5.086	0	47.441 ± 70.930	−12.902	25.812 ± 30.930	0.315	0.339	0.352	0.873	0.345
UACR (last) (mg/dL)	5.675 ± 5.086		60.343 ± 106.208		25.497 ± 30.873		0.358			

*Age, Cre, eGFR, HbA1c, Alb(U), Creatinine(U) and UACR presented as mean ± SD*.

*Empa10, Empagliflozine 10 mg/tab; Empa25, Empagliflozine 25 mg/tab; Dapa10, Dapagliflozine 10 mg/tab; Diff, difference; Vari, variate initial; Varif, variate final; Cre, creatinine; eGFR, estimated Glomerular filtration rate; HbA1c, Glycated hemoglobin; AC, ante cibum (preprandial); PC, post cibum (postprandial); AST, aspartate aminotransferase; ALT, Alanine aminotransferase; Na, sodium; K: potassium; LDL-C, low-density lipoprotein cholesterol; HDL-C, High-density lipoprotein cholesterol; TG, Triglyceride; Chol, cholesterol; TBI, total bilirubin; DBI, direct bilirubin; UACR, urine albumin to creatinine ratio*.

a *p < 0.05 between Empa10 and Empa25*,

b *p < 0.05 between Empa25 and Dapa10*,

c *p <0.05 between Empa10 and Dapa10*.

* *p < 0.05*.

AC and PC blood glucose and HbA1c levels decreased in all three groups, with a significant decrease in HbA1c levels in Dapa users compared with Empa25 users.

Regarding renal function parameters, Cre levels increased in all three groups, with a significant difference in change between Empa10 and Dapa users.

### Analysis of Renal Function Change in SGLT2 Inhibitor Users

**Figure 2:**
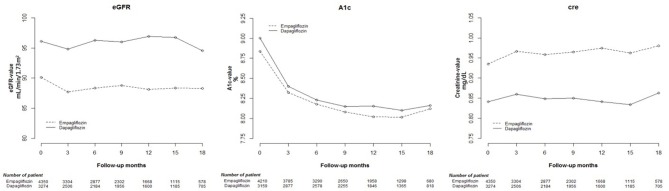
Change in estimated glomerular filtration rate (eGFR), and glycated hemoglobin (HbA1c), and creatinine (Cre) levels in empagliflozin and dapagliflozin users.

**Figure 3:**
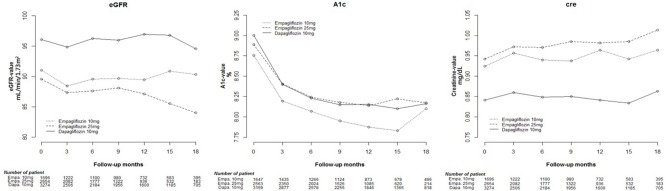
Change in estimated glomerular filtration rate (eGFR), and glycated hemoglobin (HbA1c), and creatinine (Cre) levels in empagliflozin 10 mg/tab, empagliflozin 25 mg/tab, and dapagliflozin 10 mg/tab users.

Cox proportional hazard models were used to analyze changes in eGFR, and HbA1c and Cre levels among Empa and Dapa users during follow-up (Figure 2). A lower initial eGFR and higher Cre levels were found in Empa users than in Dapa users. Regarding changes in eGFR and Cre levels over time, significant differences between the two groups were found (*p* < 0.001). We then examined changes in eGFR and Cre levels for the different SGLT2 inhibitor users. The initial Cre level was highest in Empa25 users, followed by that in Empa10 users and then in Dapa users (Table 2), that is, the opposite result to that for eGFR. Analysis of changes in eGFR and Cre levels over time revealed significant differences among the three groups (*p* < 0.001) (Figure 3).

### Analysis of eGFR Decrease Over 40% and AKI-related Hospitalization in SGLT2 Inhibitor Users

**Figure 4:**
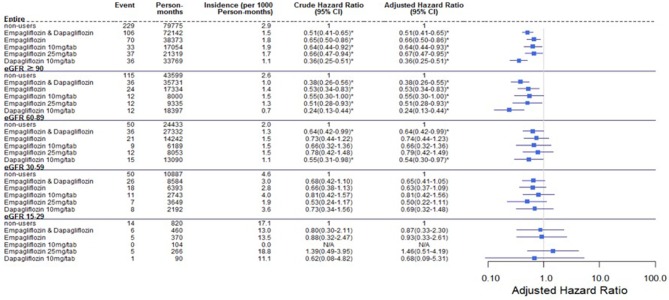
Cox proportional hazard models for decrease in eGFR over 40% in patients receiving sodium–glucose cotransporter 2 inhibitors and other drugs. The probability of a decrease in eGFR over 40% is shown for empagliflozin 10 mg/tab, empagliflozin 25 mg/tab, and dapagliflozin 10 mg/tab users vs. non-users in different renal function subgroups. eGFR, estimated glomerular filtration rate.

**Figure 5:**
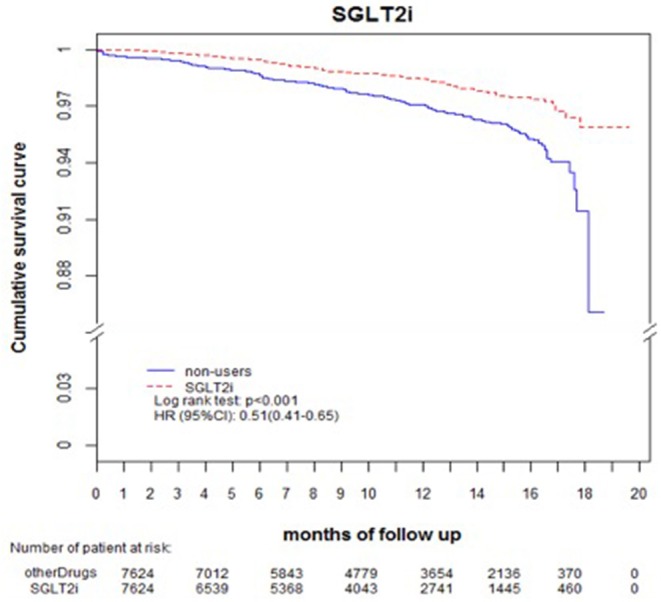
Decrease in eGFR over 40%-free survival rates in the patients with diabetes. The outcome was estimated using Cox regression models stratified according to history of eGFR decrease over 40% for SGLT2 inhibitor users vs. non-users. eGFR, estimated glomerular filtration rate; SGLT2i, sodium–glucose cotransporter 2 inhibitor.

**Figure 6:**
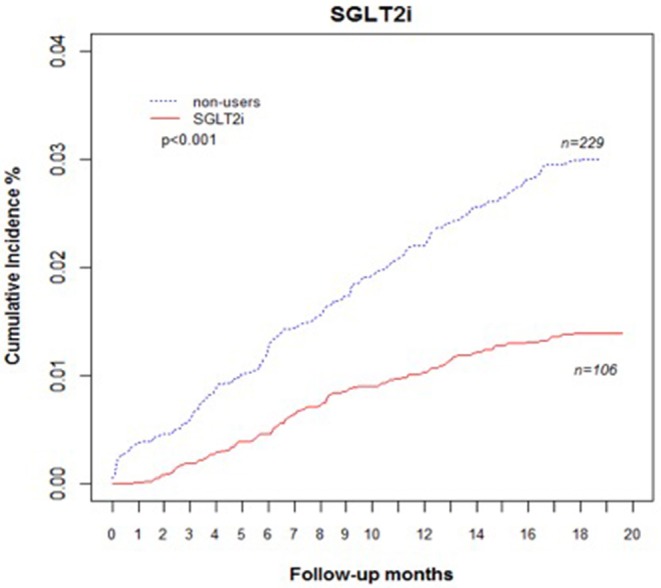
Cumulative incidence rate of decrease in eGFR over 40% in patients with diabetes. Outcomes were estimated according to history of eGFR decrease over 40% for SGLT2 inhibitor users vs. non-users. eGFR, estimated glomerular filtration rate; SGLT2i, sodium–glucose cotransporter 2 inhibitor.

Comparing eGFR decrease over 40% in SGLT2 inhibitor users and non-users, we found a lower incidence of decrease in all SGLT2 inhibitor users (HR 0.51, 95% CI 0.41–0.65) and the lowest in Dapa users (HR 0.36, 95% CI 0.25–0.51). A lower incidence of eGFR decrease over 40% in all SGLT2 inhibitor users in eGFR ≥ 90 mL/min/1.73 m^2^ and 60–89 mL/min/1.73 m^2^ subgroup (HR 0.38, 95% CI 0.26–0.55 and HR 0.64, 95% CI 0.42–0.99, respectively). But in the eGFR 60–89 mL/min/1.73 m^2^ subgroup, only Dapa users had the decreased risk (HR 0.54, 95% CI 0.30–0.97) (Table 3 and Figure 4). When followed overtime, we observed that the incidence of eGFR decrease over 40% was lower in SGLT2 inhibitor users than non-users in the 18-month follow-up (HR 0.51, 95% CI 0.41–0.65) (Figure 5). Similarly, the cumulative incidence initially increased in non-users compared with SGLT2 inhibitor users after the 18-month follow-up (*p* < 0.001) (Figure 6).

**Table 3:** Incident rate of decrease in eGFR over 40% between SGLT-2 inhibitor users and non-users in different renal function group.

**SGLT2i. Incident rate**	**Entire**			**Received Empagliflozin and Dapagliflozin**			**Non-users**		
	**Event**	**Person-months**	**Incident rate per 1,000** **Person-months(95% CI)**	**Event**	**Person-months**	**Incident rate per 1,000** **Person-months(95% CI)**	**Event**	**Person-months**	**Incident rate per 1,000** **Person-months (95% CI)**
**Entire cohort subgroup by eGFR**	335	151,916	2.21 (1.97–2.45)	106	72,142	1.47 (1.19–1.75)	229	79,775	2.87 (2.50–3.24)
≧90	151	79,330	1.90 (1.60–2.20)	36	35,731	1.01 (0.68–1.34)	115	43,599	2.64 (2.16–3.12)
60–89	86	51,765	1.66 (1.31–2.01)	36	27,332	1.32 (0.89–1.75)	50	24,433	2.05 (1.48–2.62)
30–59	76	19,471	3.90 (3.02–4.78)	26	8,584	3.03 (1.87–4.19)	50	10,887	4.59 (3.32–5.86)
15–29	20	1,280	15.62 (8.77–22.47)	6	460	13.04 (2.61–23.47)	14	820	17.07 (8.13–26.01)

**Figure 7:**
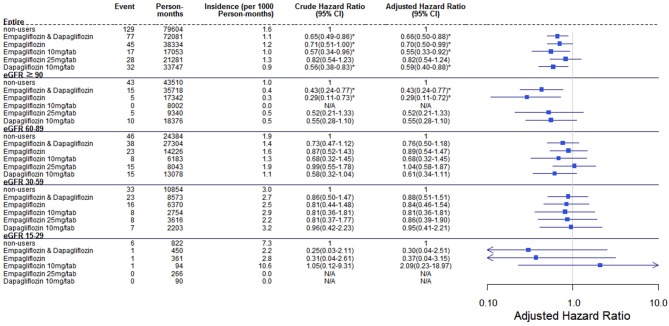
Cox proportional hazard models for AKI-related hospitalization rate in patients receiving sodium–glucose cotransporter 2 inhibitors and non-users. The probability of AKI-related hospitalization is shown for empagliflozin 10 mg/tab, empagliflozin 25 mg/tab, and dapagliflozin 10 mg/tab users vs. non-users in different renal function subgroups. AKI, acute kidney injury.

**Figure 8:**
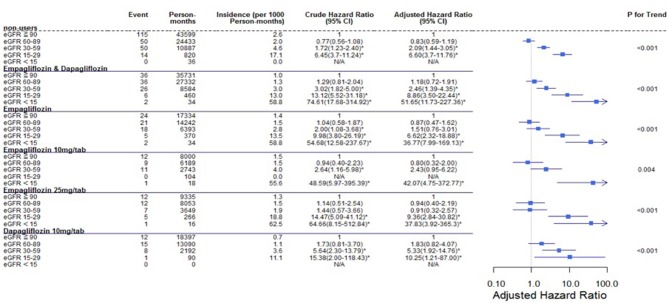
Cox proportional hazard models for AKI-related hospitalization in patients with different renal function levels. The probability of AKI-related hospitalization is shown for eGFR<15, 15–29, 30–59, and 60–89 mL/min/1.73 m^2^ vs. eGFR ≥ 90 mL/min/1.73 m^2^ in different SGLT2 inhibitor users and non-users. eGFR, estimated glomerular filtration rate; SGLT2i, sodium–glucose cotransporter 2 inhibitor.

**Figure 9:**
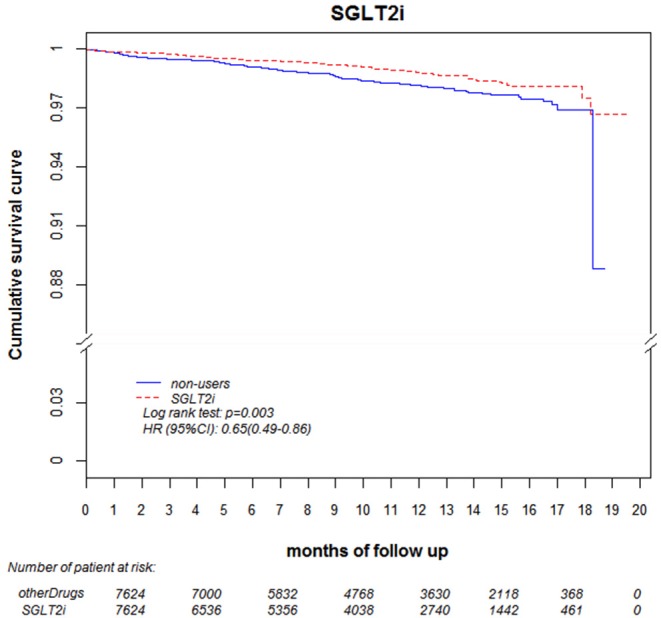
AKI-related hospitalization-free survival rates in patients with diabetes. Outcomes were estimated using Cox regression models stratified according to history of hospitalization related to AKI for SGLT2 inhibitor users vs. non-users. eGFR, estimated glomerular filtration rate; SGLT2i, sodium–glucose cotransporter 2 inhibitor; AKI, acute kidney injury.

Regarding the AKI-related hospitalization rate, we observed decreased hospitalization rates in SGLT2 inhibitor users compared with non-users (HR 0.66, 95% CI 0.50–0.88) (Figure 7). Moreover, when comparing different renal function subgroups with eGFR of ≥90 mL/min/1.73 m^2^ among the three SGLT2 inhibitor user groups and non-users, we found that the lower their eGFR, the higher was their AKI-related hospitalization rates (Figure 8). In the 18-month follow-up, there was lower AKI-related hospitalization rate observed in SGLT2 inhibitor users than non-users (HR 0.65, 95% CI 0.49–0.86) (Figure 9).

### Analysis of HbA1c Control Effect in SGLT2 Inhibitor Users

With respect to blood glucose control, a higher initial HbA1c level was found in Dapa users than in Empa users, and the trend of HbA1c change was significantly different in these two groups (Figure 2). The HbA1c change was significantly different among Dapa and Empa10 users (Figure 3). To analyze the blood glucose control effect, we set HbA1c of <7.0% as the target. We found that compared with non-users, a higher proportion of SGLT2 inhibitor users had HbA1c ≥ 7%. However, as seen in Table 1, SGLT2 inhibitor users had a lower average baseline HbA1c level and less effective HbA1c reduction compared with non-users. In general, Dapa users had the largest decreased proportion of HbA1c ≥ 7%, whereas in the eGFR 30–59 mL/min/1.73 m^2^ subgroup Empa had the better effect. In the eGFR15–29 mL/min/1.73 m^2^ subgroup, the effect of decline in the proportion of HbA1c ≥ 7% worsened, and blood glucose deteriorated as well (Supplementary Table 4). Furthermore, we analyzed the effect of blood glucose control among Empa10, Empa25, and Dapa users with differing renal function, and found that Dapa users had the largest HbA1c reduction, and Emap25 had the smallest one (Supplementary Table 5).

## Discussion

As a new class of antidiabetic drug, the SGLT2 inhibitor lowers plasma glucose levels through renal excretion (1). Because the mechanism of action of SGLT2 inhibitors is based on renal excretion of plasma glucose, whether using SGLT2 inhibitors in patients with differing renal function would increase the rate of acute renal function deterioration or hospitalization requires further discussion. Two doses of Empa, namely 10 and 25 mg/tab, became available for use at CGMH in May and July 2016, respectively, whereas another SGLT2 inhibitor subtype, dapa10, became available at CGMH in June 2016. We stratified the Empa dose to 10 or 25 mg/day according to the design of EMPA-REG Outcome Trial (3) and the latest EMPRISE study (16). According to Levine's review (17), the abilities of HbA1c decreasing between Empa10 and Empa25 as monotherapy are 1.44% vs. 1.43%, respectively. Besides, based on the previous reports, the glucose lowering effect and renal protection are similar for both Empa10 or Empa25 (18). But the real world data in different CKD stage is not well-studied. The reasons to make physician choose patients who receive Empa or Dapa are dependent on the renal function (Empa could use on the patients with eGFR > 45 mL/min/1.73 m^2^, and Dapa could use on eGFR > 60 mL/min/1.73 m^2^) and individual preference. We tried to eliminate this bias by matching initial renal function.

We therefore divided SGLT2 inhibitor users into three subgroups, namely Empa10, Empa25, and Dapa, for further comparison.

### Renal Profile

After 18-month follow-up on the renal function of SGLT2 inhibitor users and non-users, we found that Cre levels increased and eGFR reduced in both groups, with these changes being significantly lower in SGLT2 inhibitor users. Moreover, the initial pre-treatment renal function data were statistically better in SGLT2 inhibitor users compared with non-users. In a meta-analysis, Wang et al. noted that Cre levels statistically significantly increased in patients who received SGLT2 inhibitor treatment (19), whereas, Xu et al. noted significant changes in eGFR after analyzing 47 studies evaluating the effect of SGLT2 inhibitors on renal function change (9). There are multiple potential reno-protective mechanisms of SGLT2 inhibitors including decreased hyperfiltration, reduce glomerular hypertension, lower intravascular volume, etc. (5, 11, 12). Besides, there are increased composite renal events with follow-up duration between 26 and 104 weeks, but not in the other follow-up duration noted by a meta-analysis (20). This may be the reason why there are debates surrounding the effect of SGLT2 inhibitors on renal function because the exact mechanism of SGLT2 inhibitor on kidney event is still unclear and it might be time related.

Having divided SGLT2 inhibitor users into Empa users and Dapa users, we found that Empa users had lower baseline eGFR and higher Cre levels. In the 18-month follow-up period, differences in changes in eGFR and Cre levels between the two groups were statistically significant, with renal function more obviously deteriorating after 12-months in Empa users. Sugiyama et al. noted that dapagliflozin exhibited significant renoprotective effects through renal morphology, although no large increase in eGFR or decrease in Cre levels was observed (21). Since the publication of the EMPA-REG OUTCOME study, numerous studies have supported the renoprotection of Empa (6, 22). Although only a few studies have compared empagliflozin with dapagliflozin, Huilin et al. illustrated that empagliflozin resulted in fewer renal events than dapagliflozin (20), a result that differs from our findings. The different foothold of renal function in the two SGLT2 inhibitor user groups may explain this discrepancy.

Having divided SGLT2 inhibitor users into three subgroups, we found that Empa25 users had the highest baseline Cre and lowest eGFR, followed by Empa10 users with midrange values and then Dapa users, who had the lowest Cre levels and highest eGFR. After receiving SGLT2 inhibitors, the Cre levels increased in all three groups, with a statistically significant difference between Empa10 and Dapa users (0.029 vs. 0.011 mg/dL, *p* = 0.01). Overtime, the trend of changes in Cre levels and eGFR among the three groups was statistically significant (Figure 3). Specifically, Empa25 users showed deteriorating renal function after 12-months, with a statistically significant difference relative to Dapa users.

Furthermore, there was no significant difference of UACR between SGLT2 inhibitor users and non-users. Several studies have observed that SGLT2 inhibitors have a beneficial effect of UACR reduction (23), although other results for change in UACR after SGLT2 inhibitor use are more neutral (19). Despite the beneficial effect of SGLT2 inhibitors on UACR, progression to macroalbuminuria was observed in a certain percentage of patients with type 2 diabetes (6). Moreover, in our study, baseline UACR was lower in SGLT2 inhibitor users than in non-users. Among the three subgroups of SGLT2 inhibitor users, the highest UACR was found in Empa25 users, followed by Dapa and Empa10 users. After 18-month follow up, UACR increased in Empa25 users but decreased in Dapa users. Contrast to our finding, Cherney et al. demonstrated that Empa reduced UACR and that this effect was better in patients with higher baseline UACR (8). Therefore, a different foothold may influence the effect of SGLT2 inhibitors on UACR.

### Rate of eGFR Decrease Over 40% From Baseline and AKI-related Hospitalization

A lower risk of eGFR decrease over 40% was observed in eGFR ≥ 90 mL/min/1.73 m^2^ among Empa users and in eGFR ≥ 60 mL/min/1.73 m^2^ among Dapa users than non-users. Perlman et al. evaluated the relationship between SGLT2 inhibitors and acute renal failure using the U.S. Food and Drug Administration (FDA) adverse event report system database and revealed that SGLT2 inhibitors were more highly related to increased acute renal failure than other drugs (13). Nadkarni et al. found no increased risk of AKI with SGLT2 inhibitor use (24). Wanner et al. analyzed AKI in differing renal function levels, with a cut-off eGFR of 60 mL/min/1.73 m^2^ and found fewer AKI events in Empa users and patients with better renal function (6). However, Szalat et al. mentioned several possible mechanisms whereby SGLT2 inhibitors could cause acute renal failure (25).

The incidence of eGFR decrease over 40% was lower in SGLT2 inhibitor users than non-users in 18-months follow up. The benefit of reduced renal deterioration in SGLT2 inhibitor users would become apparent after a long follow-up period. Some evidence indicates that patients treated with dapagliflozin had a higher rate of recovery from deteriorated renal function and return to the baseline level than those who were treated with other drugs or discontinued dapagliflozin (20).

SGLT2 inhibitor users did not have a higher AKI-related hospitalization rate than other drug users, nor did this rate increase over time. Among the SGLT2 inhibitor user subgroups, the trend in each group was that poorer renal function in patients led to a higher AKI-related hospitalization rate (Figure 8).

### Glucose Control

Comparing SGLT2 inhibitor users with non-users, we observed a less decrease in HbA1c levels after 18-months. This might be due to the weaker glucose lowering ability and less hypoglycemia rate of SGLT2 inhibitors compared with sulfonylurea (26, 27). Besides, in real world practice, physicians may use SGLT2 inhibitors as an add-on regimen in poorly controlled patients who usually have less response to anti-diabetic drugs.

The HbA1c level decreased in all the three SGLT2 inhibitor user subgroups (Empa10, Empa25, and Dapa), with Dapa users showing the statistically significant decrease compared with Empa25 group. Moreover, a statistically significant difference in glucose change was observed among the three groups. Johnston et al. compared several studies using Empa and Dapa and discovered that Empa25 had the best glucose-lowering effect, followed by Empa10 and then Dapa (28), a finding that differs from ours. However, our data shows that the initial HbA1c level was highest in Dapa users, followed by Empa25, with Empa10 users having the lowest HbA1c level. This finding was compatible with that of Yagi et al., who noted a better glucose-lowering effect in patients with type 2 diabetes receiving SGLT2 inhibitor treatment who had a high baseline HbA1c level (29).

This study had several limitations. First, because only Empa and dapa were currently available at CGMH and were only introduced after 2016, other SGLT2 inhibitors were not studied and follow-up time was limited. Second, because this was a non-randomized, retrospective, observational study, selection bias was possible, despite comprehensive propensity score matching and our setting the index date as the start of therapy. Third, some details of renal function could not be distinguished in the diagnostic records. However, for the majority of the participants in this large cohort study, we were still able to indicate the effects of SGLT2 inhibitors. Forth, although we tried our best to eliminate the interference of other drugs between SGLT2 inhibitors users and non-users by propensity score matching, the further evaluation of the effects on drugs might be added on during the study period is limited.

## Conclusion

In real world practice, both Dapa an Empa had similar glucose-lowering effect across different CKD stages. SGLT2 inhibitor users and non-users had reduced renal function at the 18-month follow up, but SGLT2 inhibitor users exhibited lower changes compared with baseline. Moreover, SGLT2 inhibitor users had a lower incidence of eGFR decrease over 40% within 18-months without increase in the AKI-related hospitalization rate.

## Data Availability Statement

The datasets analyzed in this manuscript are not publicly available. Requests to access the datasets should be directed to C-HL, adronlin@cgmh.org.tw.

## Author Contributions

Y-HL wrote the manuscript and researched and analyzed the data. Y-YH, S-HH, J-HS, and S-TC researched the data. C-HL designed, reviewed, edited the manuscript, the guarantor of this work and, as such, had full access to all the data in the study and takes responsibility for the integrity of the data and the accuracy of the data analysis. All authors were involved in the interpretation of data, critical revision, and approval of the manuscript.

### Conflict of Interest

The authors declare that the research was conducted in the absence of any commercial or financial relationships that could be construed as a potential conflict of interest.

## References

[1] Defronzo RA Hompesch M Kasichayanula S Liu X Hong Y Pfister M. Characterization of renal glucose reabsorption in response to dapagliflozin in healthy subjects and subjects with type 2 diabetes. Diabetes Care. (2013) 36:3169–76. 10.2337/dc13-0387 23735727 PMC3781504

[2] Mikhail N. Place of sodium-glucose co-transporter type 2 inhibitors for treatment of type 2 diabetes. World J Diabetes. (2014) 5:854–9. 10.4239/wjd.v5.i6.854 25512787 PMC4265871

[3] Zinman B Wanner C Lachin JM Fitchett D Bluhmki E Hantel S. Empagliflozin, cardiovascular outcomes, and mortality in type 2 diabetes. N Eng J Med. (2015) 373:2117–28. 10.1056/NEJMoa1504720 26378978

[4] Neal B Perkovic V Mahaffey KW De Zeeuw D Fulcher G Erondu N Canagliflozin and cardiovascular and renal events in type 2 diabetes. N Engl J Med. (2017) 377:644–57. 10.1056/NEJMoa1611925 28605608

[5] Fioretto P Zambon A Rossato M Busetto L Vettor R. SGLT2 inhibitors and the diabetic kidney. Diabetes Care. (2016) 39(Suppl 2):S165–71. 10.2337/dcS15-3006 27440829

[6] Wanner C Inzucchi SE Lachin JM Fitchett D Von Eynatten M Mattheus M Empagliflozin and progression of kidney disease in type 2 diabetes. N Engl J Med. (2016) 375:323–34. 10.1056/NEJMoa1515920 27299675

[7] Wiviott SD Raz I Bonaca MP Mosenzon O Kato ET Cahn A Dapagliflozin and cardiovascular outcomes in type 2 diabetes. N Engl J Med. (2018) 380:347–35. 10.1056/NEJMoa1812389 30415602

[8] Cherney DZI Zinman B Inzucchi SE Koitka-Weber A Mattheus M Von Eynatten M. Effects of empagliflozin on the urinary albumin-to-creatinine ratio in patients with type 2 diabetes and established cardiovascular disease: an exploratory analysis from the EMPA-REG OUTCOME randomised, placebo-controlled trial. Lancet Diabetes Endocrinol. (2017) 5:610–21. 10.1016/S2213-8587(17)30182-1 28666775

[9] Xu L Li Y Lang J Xia P Zhao X Wang L. Effects of sodium-glucose co-transporter 2 (SGLT2) inhibition on renal function and albuminuria in patients with type 2 diabetes: a systematic review and meta-analysis. PeerJ. (2017) 5:e3405. 10.7717/peerj.3405 28663934 PMC5490461

[10] Liu XY Zhang N Chen R Zhao JG Yu P. Efficacy and safety of sodium-glucose cotransporter 2 inhibitors in type 2 diabetes: a meta-analysis of randomized controlled trials for 1 to 2years. J Diabetes Complicat. (2015) 29:1295–303. 10.1016/j.jdiacomp.2015.07.011 26365905

[11] Skrtic M Cherney DZ. Sodium-glucose cotransporter-2 inhibition and the potential for renal protection in diabetic nephropathy. Curr Opin Nephrol Hypertens. (2015) 24:96–103. 10.1097/MNH.0000000000000084 25470017

[12] Andrianesis V Glykofridi S Doupis J. The renal effects of SGLT2 inhibitors and a mini-review of the literature. Ther Adv Endocrinol Metab. (2016) 7:212–28. 10.1177/2042018816676239 28203358 PMC5298360

[13] Perlman A Heyman SN Matok I Stokar J Muszkat M Szalat A. Acute renal failure with sodium-glucose-cotransporter-2 inhibitors: analysis of the FDA adverse event report system database. Nutr Metab Cardiovasc Dis. (2017) 27:1108–13. 10.1016/j.numecd.2017.10.011 29174031

[14] Coresh J Turin TC Matsushita K Sang Y Ballew SH Appel LJ. Decline in estimated glomerular filtration rate and subsequent risk of end-stage renal disease and mortality. JAMA. (2014) 311:2518–31. 10.1001/jama.2014.6634 24892770 PMC4172342

[15] Levey AS Stevens LA Schmid CH Zhang YL Castro AF III Feldman HI. A new equation to estimate glomerular filtration rate. Ann Intern Med. (2009) 150:604–12. 10.7326/0003-4819-150-9-200905050-00006 19414839 PMC2763564

[16] Patorno E Pawar A Franklin JM Najafzadeh M Deruaz-Luyet A Brodovicz KG Empagliflozin and the risk of heart failure hospitalization in routine clinical care: a first analysis from the Empagliflozin Comparative Effectiveness and Safety (EMPRISE) Study. Circulation. (2019) 139:2822–30. 10.1161/CIRCULATIONAHA.118.039177 30955357 PMC6594384

[17] Levine MJ. Empagliflozin for type 2 diabetes mellitus: an overview of phase 3 clinical trials. Curr Diabetes Rev. (2017) 13:405–23. 10.2174/1573399812666160613113556 27296042 PMC5543566

[18] Heise T Seman L Macha S Jones P Marquart A Pinnetti S. Safety, tolerability, pharmacokinetics, and pharmacodynamics of multiple rising doses of empagliflozin in patients with type 2 diabetes mellitus. Diabetes Ther. (2013) 4:331–45. 10.1007/s13300-013-0030-2 23838841 PMC3889329

[19] Wang Y Hu X Liu X Wang Z. An overview of the effect of sodium glucose cotransporter 2 inhibitor monotherapy on glycemic and other clinical laboratory parameters in type 2 diabetes patients. Ther Clin Risk Manag. (2016) 12:1113–31. 10.2147/TCRM.S112236 27486328 PMC4956063

[20] Huilin T Dandan L Jingjing Z Yufeng L Tiansheng W Suodi Z Sodium-glucose co-transporter-2 inhibitors and risk of adverse renal outcomes among patients with type 2 diabetes: a network and cumulative meta-analysis of randomized controlled trials. Diabetes Obes Metab. (2017) 19:1106–15. 10.1111/dom.12917 28240446

[21] Sugiyama S Jinnouchi H Kurinami N Hieshima K Yoshida A Jinnouchi K. Impact of dapagliflozin therapy on renal protection and kidney morphology in patients with uncontrolled type 2 diabetes mellitus. J Clin Med Res. (2018) 10:466–77. 10.14740/jocmr3419w 29707088 PMC5916535

[22] Scheen AJ Delanaye P. Effects of reducing blood pressure on renal outcomes in patients with type 2 diabetes: focus on SGLT2 inhibitors and EMPA-REG OUTCOME. Diabetes Metab. (2017) 43:99–109. 10.1016/j.diabet.2016.12.010 28153377

[23] Mende CW. Diabetes and kidney disease: the role of sodium-glucose cotransporter-2 (SGLT-2) and SGLT-2 inhibitors in modifying disease outcomes. Curr Med Res Opin. (2017) 33:541–51. 10.1080/03007995.2016.1271779 27977314

[24] Nadkarni GN Ferrandino R Chang A Surapaneni A Chauhan K Poojary P. Acute kidney injury in patients on SGLT2 inhibitors: a propensity-matched analysis. Diabetes Care. (2017) 40:1479–85. 10.2337/dc17-1011 28827404 PMC5652593

[25] Szalat A Perlman A Muszkat M Khamaisi M Abassi Z Heyman SN. Can SGLT2 inhibitors cause acute renal failure? Plausible role for altered glomerular hemodynamics and medullary hypoxia. Drug Saf. (2018) 41:239–52. 10.1007/s40264-017-0602-6 28952138

[26] Nauck MA Del Prato S Meier JJ Duran-Garcia S Rohwedder K Elze M. Dapagliflozin versus glipizide as add-on therapy in patients with type 2 diabetes who have inadequate glycemic control with metformin: a randomized, 52-week, double-blind, active-controlled noninferiority trial. Diabetes Care. (2011) 34:2015–22. 10.2337/dc11-0606 21816980 PMC3161265

[27] Del Prato S Nauck M Duran-Garcia S Maffei L Rohwedder K Theuerkauf A. Long-term glycaemic response and tolerability of dapagliflozin versus a sulphonylurea as add-on therapy to metformin in patients with type 2 diabetes: 4-year data. Diabetes Obes Metab. (2015) 17:581–90. 10.1111/dom.12459 25735400

[28] Johnston R Uthman O Cummins E Clar C Royle P Colquitt J Canagliflozin, dapagliflozin and empagliflozin monotherapy for treating type 2 diabetes: systematic review and economic evaluation. Health Technol Assess. (2017) 21:1–218. 10.3310/hta21020 PMC5292646 28105986

[29] Yagi S Aihara KI Kondo T Kurahashi K Yoshida S Endo I. Predictors for the treatment effect of sodium glucose co-transporter 2 inhibitors in patients with type 2 diabetes mellitus. Adv Ther. (2018) 35:124–34. 10.1007/s12325-017-0639-z 29185199

