# Supplementary material for: Renal and Glucose-Lowering Effects of Empagliflozin and Dapagliflozin in Different Chronic Kidney Disease Stages
Source: Front Endocrinol (Lausanne). 2019 Nov 22;10:820. doi: 10.3389/fendo.2019.00820 (PMC6883723; doi:10.3389/fendo.2019.00820)
Supplement: Supplementary file 1 [file Table_1.DOCX]

Supplementary Table 1 The baseline characteristics of accounted confounding factors

| **Baseline characteristics** |  |  |  | **Post-match Standardized Difference** | **Pre-match Standardized Difference** |
| --- | --- | --- | --- | --- | --- |
|  | **SGLT2 inhibitor users** | **non-users** | ***p*-value** |  |  |
| **No.** | 7624 | 7624 | null | 7624 : 7624 | 7624 : 62837 |
| **Cre (mg/dL)** | 0.9±0.4 | 0.9±0.4 | 0.361 | 1.5% | 110.7% |
| **HbA1c (%, mmol/mol)** | 8.9±1.6  (73.8±6.03) | 9.0±2.3  (74.86±1.62) | 0.015* | 4.8% | 89.5% |
| **Follow-up period (day)** | 307.0±154.4 | 321.7±156.8 | 0.000* | 9.5% | 67.4% |
| **Sex (female), %** | 41.9% | 42.2% | 0.755 | 0.5% | 8.9% |
| **Age, year** | 61.0±11.6 | 60.6±13.4 | 0.067 | 3.2% | 52.4% |
| **DPP4 inhibitor** | 0.601 | 0.613 | 0.149 | 2.3% | 12.6% |
| **Insulin** | 19.6% | 21.2% | 0.010* | 4.2% | 11.1% |
| **GLP-1 agonist** | 2.8% | 3.0% | 0.442 | 1.3% | 8.3% |
| **SU** | 69.0% | 68.1% | 0.257 | 1.8% | 47.6% |
| **Glinide** | 2.8% | 3.1% | 0.290 | 1.8% | 23.4% |
| **Metformin** | 48.9% | 49.7% | 0.315 | 1.6% | 6.8% |
| **Statins** | 63.7% | 60.8% | 0.000* | 6.0% | 9.6% |
| **ARB** | 54.2% | 52.2% | 0.015* | 3.9% | 5.8% |
| **ACEI** | 8.5% | 8.5% | 0.862 | 0.3% | 3.0% |
| **Loop diuretics** | 5.4% | 5.0% | 0.272 | 1.7% | 30.2% |
| **Thiazides** | 5.4% | 5.4% | 0.914 | 0.2% | 16.3% |
| **Aldosterone antagonist** | 2.5% | 2.4% | 0.636 | 0.8% | 7.3% |
| **Beta Blocker** | 29.1% | 27.3% | 0.018* | 3.8% | 11.8% |
| **CCB** | 14.6% | 13.7% | 0.109 | 2.6% | 35.4% |
| **AD** | 0.0% | 0.0% | 1.000 | 0.0% | 59.2% |
| **Hypertension** | 49.3% | 52.3% | 0.000* | 6.1% | 46.1% |
| **Hyperlipidemia** | 57.7% | 61.7% | 0.000* | 8.1% | 26.3% |
| **CVA** | 1.7% | 1.7% | 1.000 | 0.0% | 68.5% |
| **Ischemia** | 1.5% | 1.5% | 0.947 | 0.1% | 65.0% |
| **Hemorrhage** | 0.2% | 0.2% | 0.739 | 0.5% | 34.6% |
| **CAD** | 12.7% | 13.2% | 0.386 | 1.4% | 21.2% |
| **Myocardial infarction** | 9.7% | 10.2% | 0.303 | 1.7% | 13.8% |
| **ischemic heart disease** | 5.5% | 5.5% | 0.972 | 0.1% | 37.2% |
| **Heart failure** | 3.1% | 3.2% | 0.677 | 0.7% | 23.1% |

Cre, HbA1c, Follow-up period and age presented as mean±SD.

Abbreviation: SGLT-2i: Sodium-glucose co-transporter 2 inhibitor; Cre: creatinine; HbA1c: Glycated hemoglobin; DPP-4: dipeptidyl peptidase-4; GLP-1: glucose-like peptide-1; SU: sulfonylurea; ARB: Angiotensin II receptor blocker; ACEI: angiotensin converting enzyme inhibitor; CCB: calcium channel blocker; AD: Alzheimer’s disease; CVA: cerebrovascular accident; CAD: coronary artery disease

*denote *p* value <0.05
