# Supplementary material for: Renal and Glucose-Lowering Effects of Empagliflozin and Dapagliflozin in Different Chronic Kidney Disease Stages
Source: Front Endocrinol (Lausanne). 2019 Nov 22;10:820. doi: 10.3389/fendo.2019.00820 (PMC6883723; doi:10.3389/fendo.2019.00820)
Supplement: Supplementary file 2 [file Table_2.DOCX]

Supplementary Table 2 Characteristics of the study population of Sodium glucose co-transporter 2 inhibitor users and nonusers.

| basic statistic |  |  |  |  | SGLT2i vs. non-users | SGLT2i vs. non-users |
| --- | --- | --- | --- | --- | --- | --- |
|  | SGLT2i | Diff(1st-last) | Matched nonusers | Diff(1st-last) | p-value | p-value Diff(1st-last) |
| AST (first before drug) (U/L) | 33.495±27.862 | 3.244 | 33.604±38.554 | 1.73 | 0.855 | 0.002 |
| AST (last) (U/L) | 30.251±27.969 |  | 31.874±33.441 |  | 0.003 |  |
| ALT (first before drug) (U/L) | 34.481±27.235 | 1.271 | 35.994±36.970 | 1.24 | 0.130 | 0.962 |
| ALT (last) (U/L) | 33.210±25.270 |  | 34.754±31.237 |  | 0.081 |  |
| Uric acid (first before drug) (mg/dL) | 5.964±2.701 | 0.047 | 5.965±1.852 | -0.019 | 0.986 | 0.012 |
| Uric acid (last) (mg/dL) | 5.917±2.740 |  | 5.984±1.867 |  | 0.266 |  |
| Na (first before drug) (mEq/L) | 138.068±13.994 | 0.061 | 139.035±7.634 | 0.041 | 0.038 | 0.913 |
| Na (last) (mEq/L) | 138.007±14.113 |  | 138.994±7.820 |  | 0.036 |  |
| K (first before drug) (mEq/L) | 4.380±2.036 | -0.001 | 4.266±0.785 | -0.059 | 0.019 | 0.014 |
| K (last) (mEq/L) | 4.381±2.036 |  | 4.325±1.002 |  | 0.269 |  |
| LDL-C (first before drug) (mg/dL) | 94.253±30.266 | -0.203 | 99.798±34.030 | 1.962 | < 0.001* | < 0.001* |
| LDL-C (last) (mg/dL) | 94.456±30.667 |  | 97.836±32.328 |  | < 0.001* |  |
| HDL-C (first before drug) (mg/dL) | 43.621±10.952 | -1.008 | 43.625±12.387 | -0.669 | 0.992 | 0.053 |
| HDL-C (last) (mg/dL) | 44.629±10.904 |  | 44.294±12.722 |  | 0.451 |  |
| TG (first before drug) (mg/dL) | 173.69±170.876 | 5.591 | 171.32±165.244 | 9.329 | 0.492 | 0.075 |
| TG (last) (mg/dL) | 168.099±168.284 |  | 161.991±149.448 |  | 0.061 |  |
| Chol (first before drug) (mg/dL) | 168.437±38.674 | -0.056 | 177.193±43.5 | 3.002 | < 0.001* | < 0.001* |
| Chol (last) (mg/dL) | 168.493±38.219 |  | 174.191±40.258 |  | < 0.001* |  |
| TBI (first before drug) (mg/dL) | 0.793±0.422 | 0 | 0.916±0.854 | 0.05 | < 0.001* | 0.008 |
| TBI (last) (mg/dL) | 0.793±0.447 |  | 0.866±0.678 |  | 0.006 |  |
| DBI (first before drug) (mg/dL) | 0.251±0.284 | -0.009 | 0.403±0.918 | 0.047 | 0.011 | 0.062 |
| DBI (last) (mg/dL) | 0.260±0.343 |  | 0.356±0.781 |  | 0.084 |  |
| Alb(U) (first before drug) (mg/L) | 106.507±258.823 | 35.96 | 219.824±1109.466 | -3.130 | 0.325 | 0.259 |
| Alb(U) (last) (mg/L) | 70.547±140.227 |  | 222.954±1110.443 |  | 0.168 |  |
| Creatinine(U) (first before drug) (mg/dL) | 100.103±59.772 | 3.674 | 120.909±75.220 | 0.527 | < 0.001* | < 0.001* |
| Creatinine(U) (last) (mg/dL) | 96.429±56.260 |  | 120.382±75.389 |  | < 0.001* |  |

AST, ALT, uric acid, Na, K, LDL-C, HDL-C, TG, Chol, TBI, DBI, Alb(U), Creatinine(U) presented as mean±SD.

Abbreviation: SGLT-2i: Sodium-glucose co-transporter 2 inhibitor; Diff: difference; AST: aspartate aminotransferase; ALT: Alanine aminotransferase; Na: sodium; K: potassium; LDL-C: low-density lipoprotein cholesterol; HDL-C: High-density lipoprotein cholesterol; TG: Triglyceride; Chol: cholesterol; TBI: total bilirubin; DBI: direct bilirubin; Alb(U): urine albumin

*denote *p* value <0.05
