# Supplementary material for: Renal and Glucose-Lowering Effects of Empagliflozin and Dapagliflozin in Different Chronic Kidney Disease Stages
Source: Front Endocrinol (Lausanne). 2019 Nov 22;10:820. doi: 10.3389/fendo.2019.00820 (PMC6883723; doi:10.3389/fendo.2019.00820)
Supplement: Supplementary file 3 [file Table_3.DOCX]

Supplementary Table 3 Characteristics of the study population of three kinds of Sodium glucose co-transporter 2 inhibitor

| **basic statistic** | **Empagliflozin**  **(10 mg)** | **Diff**  **(1st-last)** | **Empaglifozin**  **(25 mg)** | **Diff**  **(1st-last)** | **Dapagliflozin**  **(10mg)** | **Diff**  **(1st-last)** | **Empa10 vs Empa25 vs Dapa**  ***p* value** | **Empa10 vs Empa25**  **diff (varl-varf)**  ***p* value** | **Empa10**  **vs**  **Dapa**  **diff (varl-varf)**  ***p* value** | **Empa25**  **vs**  **Dapa**  **diff(varl-varf)**  ***p* value** |
| --- | --- | --- | --- | --- | --- | --- | --- | --- | --- | --- |
| **AC glucose (first before drug) (mg/dL, mmol/L)** | 167.364±56.036  (9.3±3.1) | 24.048  (1.3) | 167.043±61.257  (9.3±3.4) | 22.803  (1.3) | 173.551±54.997  (9.6±3.1) | 25.836  (1.4) | < 0.001^b,c^ | 0.486 | 0.279 | 0.052 |
| **AC glucose (last)**  **(mg/dL, mmol/L)** | 143.316±47.674  (8.0±2.6) |  | 144.240±50.449  (8.0±2.8) |  | 147.715±48.374  (8.2±2.7) |  | 0.005^b,c^ |  |  |  |
| **PC glucose (first before drug) (mg/dL, mmol/L)** | 234.538±92.881  (13.0±5.2) | 26.057  (1.4) | 223.852±97.649  (12.4±5.4) | 9.879  (0.5) | 228.877±85.776  (12.7±4.8) | 16.717  (0.9) | 0.448 | 0.006* | 0.133 | 0.211 |
| **PC glucose (last)**  **(mg/dL, mmol/L)** | 208.481±84.759  (11.6±4.7) |  | 213.973±99.370  (11.9±5.5) |  | 212.16±91.312  (11.8±5.1) |  | 0.798 |  |  |  |
| **AST (first before drug) (U/L)** | 33.160±26.725 | 3.709 | 32.359±26.989 | 3.355 | 34.512±29.185 | 3.897 | 0.023^b^ | 0.615 | 0.784 | 0.285 |
| **AST (last) (U/L)** | 29.451±23.578 |  | 29.004±24.411 |  | 30.615±26.428 |  | 0.067 |  |  |  |
| **ALT (first before drug) (U/L)** | 32.600±23.368 | 0.812 | 34.49±29.043 | 2.429 | 34.264±23.971 | 1.2 | 0.495 | 0.175 | 0.711 | 0.163 |
| **ALT (last) (U/L)** | 31.788±20.240 |  | 32.061±24.19 |  | 33.064±24.028 |  | 0.637 |  |  |  |
| **Uric acid (first before drug) (mg/dL)** | 5.918±1.656 | 0.085 | 6.047±1.732 | 0.04 | 5.903±3.562 | 0.084 | 0.417 | 0.472 | 0.993 | 0.272 |
| **Uric acid (last) (mg/dL)** | 5.833±1.892 |  | 6.007±1.702 |  | 5.819±3.559 |  | 0.226 |  |  |  |
| **Na (first before drug) (mEq/L)** | 139.334±9.968 | 0.622 | 135.341±20.029 | 0.714 | 139.106±8.512 | -0.054 | < 0.001^a,b^ | 0.901 | 0.300 | 0.095 |
| **Na (last) (mEq/L)** | 138.712±11.498 |  | 134.627±21.094 |  | 139.160±9.154 |  | < 0.001^a,b^ |  |  |  |
| **K (first before drug) (mEq/L)** | 4.359±0.654 | -0.102 | 4.380±0.655 | 0.017 | 4.425±3.324 | -0.013 | 0.845 | 0.203 | 0.337 | 0.054 |
| **K (last) (mEq/L)** | 4.461±2.082 |  | 4.363±0.656 |  | 4.438±3.325 |  | 0.728 |  |  |  |
| **LDL (first before drug) (mg/dL)** | 94.887±30.348 | -0.018 | 92.444±30.609 | 0.57 | 95.453±29.693 | -0.253 | 0.004^a,b^ | 0.451 | 0.754 | 0.211 |
| **LDL (last) (mg/dL)** | 94.905±30.314 |  | 91.874±30.878 |  | 95.706±29.887 |  | < 0.001^a,b^ |  |  |  |
| **HDL (first before drug) (mg/dL)** | 43.863±10.893 | -1.514 | 43.820±10.954 | -0.838 | 43.338±11.389 | -0.959 | 0.724 | 0.018* | 0.057 | 0.644 |
| **HDL (last) (mg/dL)** | 45.377±11.081 |  | 44.658±10.803 |  | 44.297±11.383 |  | 0.309 |  |  |  |
| **Trig (first before drug) (mg/dL)** | 172.698±147.632 | 7.316 | 178.993±180.776 | 6.272 | 172.900±190.331 | 3.57 | 0.442 | 0.743 | 0.244 | 0.285 |
| **Trig (last) (mg/dL)** | 165.382±143.750 |  | 172.721±165.404 |  | 169.330±198.067 |  | 0.491 |  |  |  |
| **Chol (first before drug) (mg/dL)** | 168.649±39.023 | -0.139 | 167.916±40.225 | -0.398 | 169.457±37.803 | 0.166 | 0.410 | 0.727 | 0.660 | 0.302 |
| **Chol (last) (mg/dL)** | 168.788±38.128 |  | 168.314±40.27 |  | 169.291±37.655 |  | 0.696 |  |  |  |
| **TBI (first before drug) (mg/dL)** | 0.762±0.484 | 0.008 | 0.858±0.523 | -0.017 | 0.739±0.369 | 0.007 | 0.016^b^ | 0.118 | 0.928 | 0.100 |
| **TBI (last) (mg/dL)** | 0.754±0.494 |  | 0.875±0.588 |  | 0.732±0.378 |  | 0.005^a,b^ |  |  |  |
| **DBI (first before drug) (mg/dL)** | 0.300±0.171 | 0 | 0.299±0.442 | -0.03 | 0.212±0.116 | 0.001 | 0.304 | 0.115 | 0.785 | 0.109 |
| **DBI (last) (mg/dL)** | 0.300±0.171 |  | 0.329±0.548 |  | 0.211±0.114 |  | 0.267 |  |  |  |
| **Alb (U) (first before drug) (mg/dL)** | 7.704±5.370 | 0.057 | 105.729±328.853 | 75.236 | 89.135±150.408 | 14.605 | 0.648 | 0.303 | 0.178 | 0.408 |
| **Alb (U) (last) (mg/dL)** | 7.647±5.265 |  | 30.493±56.314 |  | 74.530±145.716 |  | 0.230 |  |  |  |
| **Creatinine(U) (first before drug) (mg/dL)** | 101.950±59.464 | 3.621 | 94.800±56.883 | 4.844 | 98.286±58.202 | 1.788 | 0.032^a^ | 0.288 | 0.064 | 0.001* |
| **Creatinine(U) (last) (mg/dL)** | 98.329±56.092 |  | 89.956±50.432 |  | 96.498±56.876 |  | 0.001^a,b^ |  |  |  |

AC glucose, PC glucose, AST, ALT, uric acid, Na, K, LDL-C, HDL-C, TG, Chol, TBI, DBI, Alb(U), Creatinine(U) and UACR presented as mean±SD.

Abbreviation: Empa10: Empagliflozine 10mg/tab; Empa25: Empagliflozine 25mg/tab; Dapa10: Dapagliflozine 10mg/tab; Diff: difference; Vari: variate initial; Varif: variate final; AC: ante cibum (preprandial) ; PC: post cibum (postprandial); AST: aspartate aminotransferase; ALT: Alanine aminotransferase; Na: sodium; K: potassium; LDL-C: low-density lipoprotein cholesterol; HDL-C: High-density lipoprotein cholesterol; TG: Triglyceride; Chol: cholesterol; TBI: total bilirubin; DBI: direct bilirubin; Alb(U): urine albumin

^a^ denotes *p*<0.05 between Empa10 and Empa25, ^b^ denotes *p*<0.05 between Empa25 and Dapa10, ^c^ denotes *p*<0.05 between Empa10 and Dapa10

*denotes *p*<0.05
