# Supplementary material for: Renal and Glucose-Lowering Effects of Empagliflozin and Dapagliflozin in Different Chronic Kidney Disease Stages
Source: Front Endocrinol (Lausanne). 2019 Nov 22;10:820. doi: 10.3389/fendo.2019.00820 (PMC6883723; doi:10.3389/fendo.2019.00820)
Supplement: Supplementary file 4 [file Table_4.DOCX]

Supplementary Table 4 The odds of HbA1c ≧7% (53 mmol/mol) in SGLT-2 inhibitor users compared with non-users in different renal function group

| **First_A1c~Patients &Last_A1c~Patients** | **First_A1c ~ Patients** | | **Last_A1c ~ Patients** | |
| --- | --- | --- | --- | --- |
|  | **A1C (>=7.0% vs. <7.0%)** | | **A1C (>=7.0% vs. <7.0%)** | |
|  | **OR (95%CI)** | **P-value** | **OR (95%CI)** | **P-value** |
| **Entire** |  |  |  |  |
| SGLT2 inhibitor non-user | 1 |  | 1 |  |
| Empagliflozin & Dapagliflozin | 3.29 (2.98,3.64) | <0.001* | 2.21 (2.04,2.38) | < 0.001* |
| Empagliflozin | 2.61 (2.33,2.93) | <0.001* | 2.00 (1.82,2.19) | < 0.001* |
| [Empagliflozin 10mg/tab](http://cghasp.cgmh.org.tw/newmedic/medic_result.asp?sqno=&t_dgno=PME122M&t_dgnm=Empagliflozin%2010mg/tab&t_dgmrcnm=Jardiance%2010mg%20%AE%A6%B1%C6%BF%7d%BD%A4%A6%E7%BF%F5) | 2.42 (2.06,2.86) | <0.001* | 1.67 (1.47,1.89) | < 0.001* |
| Empagliflozin 25mg/tab | 2.75 (2.38,3.17) | < 0.001* | 2.26 (2.02,2.54) | < 0.001* |
| Dapagliflozin 10mg/tab | 4.92 (4.19,5.78) | < 0.001* | 2.54 (2.29,2.83) | < 0.001* |
| **eGFR ≧90 mL/min/1.73m^2^** |  |  |  |  |
| SGLT2 inhibitor non-user | 1 |  | 1 |  |
| Empagliflozin & Dapagliflozin | 3.25 (2.82,3.76) | < 0.001* | 2.01 (1.81,2.23) | < 0.001* |
| Empagliflozin | 2.47 (2.09,2.92) | < 0.001* | 1.77 (1.55,2.01) | < 0.001* |
| [Empagliflozin 10mg/tab](http://cghasp.cgmh.org.tw/newmedic/medic_result.asp?sqno=&t_dgno=PME122M&t_dgnm=Empagliflozin%2010mg/tab&t_dgmrcnm=Jardiance%2010mg%20%AE%A6%B1%C6%BF%7d%BD%A4%A6%E7%BF%F5) | 2.51 (1.96,3.22) | < 0.001* | 1.55 (1.30,1.85) | < 0.001* |
| Empagliflozin 25mg/tab | 2.44 (1.98,3.00) | < 0.001* | 1.94 (1.65,2.28) | < 0.001* |
| Dapagliflozin 10mg/tab | 4.94 (3.94,6.19) | < 0.001* | 2.35 (2.04,2.71) | < 0.001* |
| **eGFR 60-89 mL/min/1.73m^2^** |  |  |  |  |
| SGLT2 inhibitor non-user | 1 |  | 1 |  |
| Empagliflozin & Dapagliflozin | 3.93 (3.32,4.64) | < 0.001* | 2.60 (2.28,2.96) | < 0.001* |
| Empagliflozin | 3.05 (2.52,3.70) | < 0.001* | 2.33 (2.00,2.72) | < 0.001* |
| [Empagliflozin 10mg/tab](http://cghasp.cgmh.org.tw/newmedic/medic_result.asp?sqno=&t_dgno=PME122M&t_dgnm=Empagliflozin%2010mg/tab&t_dgmrcnm=Jardiance%2010mg%20%AE%A6%B1%C6%BF%7d%BD%A4%A6%E7%BF%F5) | 2.78 (2.12,3.65) | < 0.001* | 1.97 (1.59,2.44) | < 0.001* |
| Empagliflozin 25mg/tab | 3.25 (2.57,4.12) | < 0.001* | 2.61 (2.16,3.16) | < 0.001* |
| Dapagliflozin 10mg/tab | 5.97 (4.59,7.76) | < 0.001* | 3.01 (2.52,3.60) | < 0.001* |
| **eGFR 30-59 mL/min/1.73m^2^** |  |  |  |  |
| SGLT2 inhibitor non-user | 1 |  | 1 |  |
| Empagliflozin & Dapagliflozin | 2.45 (1.88,3.19) | < 0.001* | 2.22 (1.77,2.77) | < 0.001* |
| Empagliflozin | 2.41 (1.81,3.22) | < 0.001* | 2.11 (1.66,2.69) | < 0.001* |
| [Empagliflozin 10mg/tab](http://cghasp.cgmh.org.tw/newmedic/medic_result.asp?sqno=&t_dgno=PME122M&t_dgnm=Empagliflozin%2010mg/tab&t_dgmrcnm=Jardiance%2010mg%20%AE%A6%B1%C6%BF%7d%BD%A4%A6%E7%BF%F5) | 1.82 (1.23,2.69) | 0.003 | 1.52 (1.10,2.11) | 0.012 |
| Empagliflozin 25mg/tab | 2.96 (2.05,4.28) | < 0.001* | 2.67 (1.97,3.63) | < 0.001* |
| Dapagliflozin 10mg/tab | 2.58 (1.60,4.14) | < 0.001* | 2.62 (1.74,3.94) | < 0.001* |
| **eGFR 15-29 mL/min/1.73m^2^** |  |  |  |  |
| SGLT2 inhibitor non-user | 1 |  | 1 |  |
| Empagliflozin & Dapagliflozin | 2.72 (0.86,8.57) | 0.088 | 3.02 (1.15,7.92) | 0.025 |
| Empagliflozin | 2.21 (0.70,7.02) | 0.179 | 2.99 (1.06,8.44) | 0.038 |
| [Empagliflozin 10mg/tab](http://cghasp.cgmh.org.tw/newmedic/medic_result.asp?sqno=&t_dgno=PME122M&t_dgnm=Empagliflozin%2010mg/tab&t_dgmrcnm=Jardiance%2010mg%20%AE%A6%B1%C6%BF%7d%BD%A4%A6%E7%BF%F5) | 2.27 (0.27,18.91) | 0.450 | 1.05 (0.26,4.27) | 0.945 |
| Empagliflozin 25mg/tab | 2.19 (0.60,8.04) | 0.237 | 5.91 (1.32,26.52) | 0.020 |
| Dapagliflozin 10mg/tab | 9643490.8 (0,Inf) | 0.990 | 3.15 (0.38,26.46) | 0.290 |
