# Supplementary material for: Renal and Glucose-Lowering Effects of Empagliflozin and Dapagliflozin in Different Chronic Kidney Disease Stages
Source: Front Endocrinol (Lausanne). 2019 Nov 22;10:820. doi: 10.3389/fendo.2019.00820 (PMC6883723; doi:10.3389/fendo.2019.00820)
Supplement: Supplementary file 5 [file Table_5.DOCX]

Supplementary Table 5 The HbA1c change percentage in Empagliflozin 10mg/tab, Empagliflozin 25mg/tab and Dapagliflozine 10mg/tab

| **HbA1c change percentage (mmol/mol)** | **Empagliflozin 10mg/tab** | **Empagliflozin 25mg/tab** | **Dapagliflozin 10mg/tab** | **Empa10 vs Empa25** | **Empa25 vs Dapa** | **Empa10 vs Dapa** | **Empa10 v.s Empa25 v.s Dapa** |
| --- | --- | --- | --- | --- | --- | --- | --- |
|  | **Percentage (%)** | **Percentage (%)** | **Percentage (%)** | ***p*-value** | ***p*-value** | ***p*-value** | ***p*-value** |
| **Entire** |  |  |  |  |  |  |  |
| DiffA1C ＜0.3 (3.28) | 44.3 | 47.0 | 41.1 | 0.094 | < 0.001* | 0.030* | < 0.001* |
| DiffA1C 0.3-0.6 (3.28-6.56) | 9.5 | 11.3 | 10.1 | 0.067 | 0.150 | 0.527 | 0.130 |
| DiffA1C 0.6-1.0 (6.56-10.93) | 10.6 | 10.8 | 11.5 | 0.835 | 0.438 | 0.352 | 0.551 |
| DiffA1C 1.0-1.5 (10.93-16.34) | 12.5 | 11.4 | 13.5 | 0.281 | 0.019* | 0.381 | 0.059 |
| DiffA1C ＞1.5 (16.34) | 23.0 | 19.4 | 23.8 | 0.005* | < 0.001* | 0.563 | < 0.001* |
| **eGFR ≧90** |  |  |  |  |  |  |  |
| DiffA1C ＜0.3 (3.28) | 40.0 | 41.1 | 38.7 | 0.645 | 0.209 | 0.583 | 0.429 |
| DiffA1C 0.3-0.6 (3.28-6.56) | 9.5 | 12.6 | 9.3 | 0.036* | 0.004* | 0.911 | 0.008* |
| DiffA1C 0.6-1.0 (6.56-10.93) | 10.5 | 11.9 | 11.9 | 0.372 | 1.000 | 0.330 | 0.545 |
| DiffA1C 1.0-1.5 (10.93-16.34) | 12.5 | 11.3 | 13.7 | 0.462 | 0.056 | 0.417 | 0.141 |
| DiffA1C ＞1.5 (16.34) | 27.6 | 23.1 | 26.4 | 0.026* | 0.048* | 0.550 | 0.046* |
| **eGFR 60-89** |  |  |  |  |  |  |  |
| DiffA1C ＜0.3 (3.28) | 46.9 | 50.6 | 42.5 | 0.170 | < 0.001* | 0.081 | 0.001* |
| DiffA1C 0.3-0.6 (3.28-6.56) | 9.8 | 11.5 | 11.3 | 0.341 | 0.913 | 0.395 | 0.556 |
| DiffA1C 0.6-1.0 (6.56-10.93) | 11.3 | 10.8 | 11.3 | 0.804 | 0.725 | 1.000 | 0.906 |
| DiffA1C 1.0-1.5 (10.93-16.34) | 13.0 | 11.6 | 13.7 | 0.473 | 0.170 | 0.728 | 0.355 |
| DiffA1C ＞1.5 (16.34) | 19.0 | 15.5 | 21.2 | 0.085 | 0.001* | 0.290 | 0.003 |
| **eGFR 30-59** |  |  |  |  |  |  |  |
| DiffA1C ＜0.3 (3.28) | 51.1 | 55.3 | 51.8 | 0.319 | 0.440 | 0.956 | 0.494 |
| DiffA1C 0.3-0.6 (3.28-6.56) | 9.1 | 8.1 | 10.8 | 0.760 | 0.319 | 0.631 | 0.523 |
| DiffA1C 0.6-1.0 (6.56-10.93) | 8.7 | 8.4 | 9.5 | 0.979 | 0.740 | 0.899 | 0.892 |
| DiffA1C 1.0-1.5 (10.93-16.34) | 12.1 | 10.8 | 10.4 | 0.690 | 0.957 | 0.641 | 0.805 |
| DiffA1C ＞1.5 (16.34) | 18.9 | 17.4 | 17.6 | 0.674 | 1.000 | 0.786 | 0.864 |
| **eGFR 15-29** |  |  |  |  |  |  |  |
| DiffA1C ＜0.3 (3.28) | 45.5 | 43.8 | 44.4 | 1.000 | 1.000 | 1.000 | 0.995 |
| DiffA1C 0.3-0.6 (3.28-6.56) | 9.1 | 3.1 | 11.1 | 1.000 | 0.915 | 1.000 | 0.575 |
| DiffA1C 0.6-1.0 (6.56-10.93) | 18.2 | 9.4 | 11.1 | 0.810 | 1.000 | 1.000 | 0.732 |
| DiffA1C 1.0-1.5 (10.93-16.34) | 9.1 | 18.8 | 11.1 | 0.783 | 0.971 | 1.000 | 0.691 |
| DiffA1C ＞1.5 (16.34) | 18.2 | 25.0 | 22.2 | 0.962 | 1.000 | 1.000 | 0.896 |

Abbreviation: HbA1c: glycosylated hemoglobulin; eGFR: estimated glomerular filtration rate; Empa10: Empagliflozine 10mg/tab; Empa25: Empagliflozine 25mg/tab; Dapa10: Dapagliflozine 10mg/tab; Diff: difference

*denotes *p* value <0.05
